# Peroxisomes as emerging clinical targets in neuroinflammatory diseases

**DOI:** 10.3389/fnmol.2025.1642590

**Published:** 2025-08-29

**Authors:** Andrej Roczkowsky, Richard A. Rachubinski, Tom C. Hobman, Christopher Power

**Affiliations:** ^1^Department of Medicine, University of Alberta, Edmonton, AB, Canada; ^2^Department of Cell Biology, University of Alberta, Edmonton, AB, Canada; ^3^Department of Medical Microbiology and Immunology, University of Alberta, Edmonton, AB, Canada

**Keywords:** peroxisome, peroxin, neuroinflammation, virus, neurodegeneration

## Abstract

Peroxisomes are membrane-bounded organelles that contribute to a range of physiological functions in eukaryotic cells. In the central nervous system (CNS), peroxisomes are implicated in several vital homeostatic functions including, but not limited to, reactive oxygen species signaling and homeostasis; generation of critical myelin sheath components (including ether phospholipids); biosynthesis of neuroprotective docosahexaenoic acid; breakdown of neurotoxic metabolites (such as very-long chain fatty acids); and, intriguingly, glial activation and response to inflammatory stimuli. Indeed, peroxisomes play a critical role in modulating inflammatory responses and are key regulators of the mitochondrial antiviral signaling (MAVS) protein-mediated response to infections. The importance of peroxisomes in CNS physiology is exemplified by the peroxisome biogenesis disorders (PBDs), a spectrum of inherited disorders of peroxisome assembly and/or abundance, that are characterized in part by neurological manifestations ranging from severe cerebral malformations to vision and hearing loss, depending on the individual disorder. Recently, peroxisome dysfunction has been implicated in neurological diseases associated with neuroinflammation including Alzheimer’s disease, amyotrophic lateral sclerosis, multiple sclerosis, and Parkinson’s disease while also contributing to the pathogenesis of neurotropic viruses including SARS-CoV-2, Human Pegivirus, HIV-1 and Zika virus. In the present review, we examine the diverse roles that peroxisomes serve in CNS health before reviewing more recent studies investigating peroxisome dysfunction in inflammatory brain disorders and also highlight potential peroxisomal targets for diagnostic biomarkers and therapeutic interventions.

## Introduction

Peroxisomes are membrane-bounded organelles so named for their ability to both generate and degrade hydrogen peroxide via enzymes contained in their matrix (Kumar et al., 2024). Since their initial discovery 70 years ago, peroxisomes have been implicated in a range of vital physiological functions in eukaryotic cells, including β-oxidation of very-long chain fatty acids (VLCFAs), biosynthesis of ether phospholipids, and metabolism of reactive oxygen species (ROS) (Smith and Aitchison, 2013). Given their diverse functions, it is unsurprising that the enzymatic contents and abundance of peroxisomes are highly dynamic and can change depending on cellular stressors and cues from the environment (Smith and Aitchison, 2013). Although ubiquitously found throughout the human body apart from red blood cells, peroxisomes perform several specialized functions in the central nervous system (CNS), including the synthesis of critical myelin sheath lipids, modulation of microglial activation, and glutamate metabolism (Berger et al., 2016). The importance of peroxisomes in CNS homeostasis is highlighted by congenital neurological disorders driven by aberrant peroxisome biogenesis (e.g., the peroxisome biogenesis disorders or PBDs) that are associated with impaired neuronal migration, loss of vision and hearing, seizures and reduced life expectancy, depending on the specific genetic mutation (Berger et al., 2016; Uzor et al., 2020).

**Figure 1:**
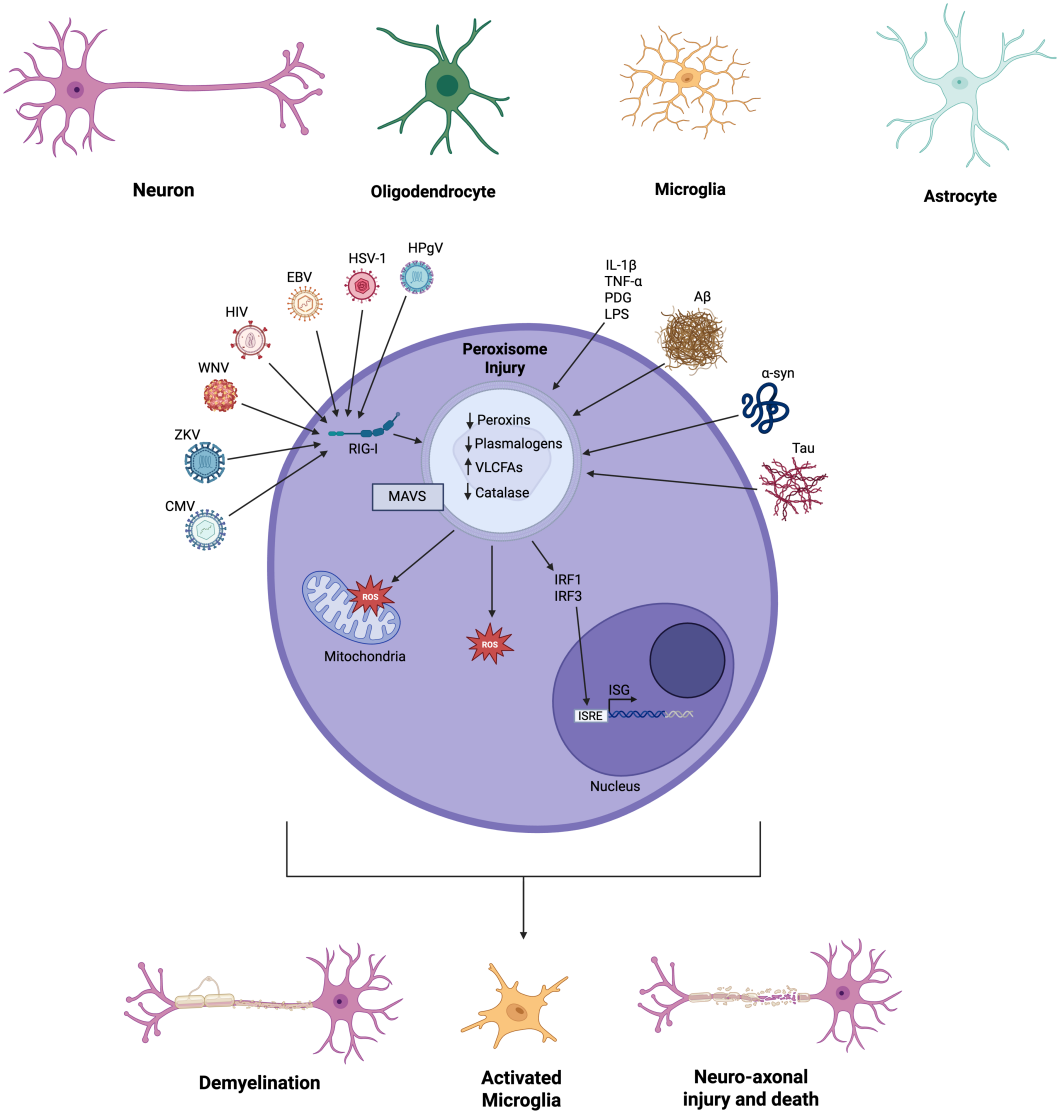
Peroxisome expression and functions in neural cells. All cell types (e.g., neurons, microglia, astrocytes, and oligodendrocytes) in the central nervous system contain peroxisomes, which exert a range of effects including production and scavenging ROS, uptake of very long chain fatty acids (VLFAs), production of interferon regulatory proteins, fatty acid synthesis, and plasmalogen production. Perturbations in peroxisome function and numbers caused by specific molecules (e.g., cytokines (IL-1β, TNF-α), bacterial lipopolysaccharide (LPS) and peptidoglycan (PGN), amyloid-beta (Aβ), alpha-synuclein (α-syn), and Tau) and neurotropic viruses [e.g., Herpes Simplex-1 (HSV-1), Epstein–Barr (EBV), Cytomegalovirus (CMV), Zika (ZKV), Human Pegivirus (HPgV), West Nile (WNV), and Human Immunodeficiency (HIV) Viruses] can lead to diverse pathogenic outcomes including glial activation, demyelination, neuro-axonal damage and cell death (created in BioRender).

Recently, peroxisomes have been identified as key modulators of the innate immune response (Di Cara, 2020; Di Cara et al., 2019). In response to viral infections, peroxisome-localized mitochondrial antiviral signaling protein (MAVS) is activated, leading to downstream production of type I and III interferons and cytokines (Dixit et al., 2010; Bender et al., 2015). Several neurotropic viruses such as HIV-1 and Zika virus have evolved mechanisms to suppress peroxisomes and their activities, thereby dampening the antiviral response in glia (Xu et al., 2017; Wong et al., 2019). Peroxisomes have also been implicated in the antibacterial innate immune response via NF-κB signaling pathways and play an essential role in phagocytosis (Di Cara et al., 2017). Within the CNS, peroxisomes modulate the activation and phagocytic abilities of microglia via their roles in lipid metabolism and the production of anti-inflammatory long-chain polyunsaturated fatty acids such as eicosapentaenoic acid (EPA) and docosahexaenoic acid (DHA) (Di Cara et al., 2019; Hjorth and Freund-Levi, 2012). Given their key role in the CNS and their immunomodulatory properties, it is unsurprising that peroxisomes have been implicated in the pathogenesis several neuroinflammatory and other neurological disorders with an inflammatory component, such as multiple sclerosis (Roczkowsky et al., 2022; Gray et al., 2014), Alzheimer’s disease (Semikasev et al., 2023; Kou et al., 2011), amyotrophic lateral sclerosis (ALS) (Jo and Cho, 2019), Parkinson’s disease (Cipolla and Lodhi, 2017), and contributing to the pathogenesis of several neurotropic viruses (e.g., SARS-CoV-2, HIV-1, Human Pegivirus, Zika virus, West Nile, virus, Yellow fever virus, and Epstein–Barr virus) (Xu et al., 2017; Wong et al., 2019; Roczkowsky et al., 2023; Doan et al., 2021). In the present review, we explore the roles of peroxisomes in neuroinflammation (Figure 1) and discuss the evidence supporting peroxisomes as therapeutic targets in these neurological conditions and viral infections.

## Peroxisome biology and function

The biogenesis of peroxisomes is tightly regulated by a family of proteins known as the peroxins (also termed peroxisome biogenesis factors) encoded by 14 human *PEX* genes (Smith and Aitchison, 2013; Farré et al., 2019). Peroxisome abundance is increased via two principal pathways: (1) growth and division of existing peroxisomes and (2) *de novo* biogenesis of peroxisomes. Growth and division begin with elongation of existing cellular peroxisomes that is initiated by insertion of PEX11 isoforms into the peroxisome membrane. Several adaptor proteins are then recruited to the peroxisome membrane culminating in the assembly of a Dynamin-Related Protein 1 (DRP1) ring around the elongated peroxisome for scission to occur (the final step of fission). *De novo* biogenesis of peroxisomes is initiated by the insertion of peroxisomal membrane proteins into a specialized region of the endoplasmic reticulum (ER) called the preperoxisomal ER. Preperoxisomal vesicles (ppVs) containing a subset of peroxisomal membrane proteins then bud from this region of the ER and go on to fuse with other ppVs or mature peroxisomes. Interestingly, a subpopulation of ppVs arise from the mitochondria rather than the ER, although it is currently unknown if this subset of peroxisomes is functionally different from solely ER-derived peroxisomes (Fransen et al., 2017).

Peroxins also play a critical role in the targeting of peroxisomal membrane and matrix proteins to the peroxisome (Smith and Aitchison, 2013; Farré et al., 2019). Soluble proteins destined for the peroxisomal interior are translated in the cytosol and contain peroxisome targeting signals (PTS1 and PTS2) marking them for uptake into the peroxisome matrix, while peroxisomal membrane proteins contain membrane protein targeting signals (mPTSs). Protein trafficking to the peroxisome and uptake into the peroxisomal matrix are facilitated by PEX5 and PEX7, which bind PTS1 and PTS2, respectively, in the cytosol (Kumar et al., 2024; Farré et al., 2019; Baker et al., 2016). PEX5 and PEX7 then interact with the importomer complex consisting of several peroxins including PEX2, PEX10, PEX12, PEX13 and PEX14, resulting in protein uptake into the peroxisomal matrix (Meinecke et al., 2010). Proteins are incorporated into the peroxisome membrane via binding of their mPTS by PEX19, which subsequently docks with PEX3 at the ER or on the peroxisome membrane to facilitate the specific targeting of peroxisomal membrane proteins (Smith and Aitchison, 2013; Farré et al., 2019).

Peroxisome degradation and turnover occur in part through a form of selective autophagy called pexophagy (Germain and Kim, 2020). Ubiquitination of peroxisomal proteins, including PEX5 and PMP70 (encoded by the *ABCD3* gene), by the E3 ubiquitin ligase activity of PEX2 targets peroxisomes for pexophagy (Sargent et al., 2016). Under physiological conditions, peroxisome biogenesis and degradation are in dynamic equilibrium, as ubiquitin can also be removed from peroxisomal proteins by the deubiquitinase USP30 and the peroxisomal AAA-ATPases PEX1 and PEX6 (Germain and Kim, 2020). Ubiquitin proteins p62 and NBR1 act as pexophagy receptors, which recruit and interact with the autophagosome complex through interactions with LC3-II (Ichimura et al., 2008; Kirkin et al., 2009). Multiple stimuli are known to induce pexophagy in mammalian cells, including ROS, amino acid starvation and hypoxia (Germain and Kim, 2020).

Although initially characterized and named for their ability to generate and degrade hydrogen peroxide, peroxisomes contribute to an array of cellular metabolic functions (Wanders et al., 2023). Peroxisomes contain enzymatic machinery for fatty acid oxidation (both α- and β-oxidation) and contribute to the metabolism of a subset of fatty acid species that cannot be processed by mitochondria alone, including VLCFAs (≥22 carbons) and certain branched chain fatty acids (Lodhi and Semenkovich, 2014). Although the peroxisomal fatty acid oxidation system appears to contribute little to cellular ATP production, it plays a major role in the oxidation of toxic fatty acid species, such as certain VLCFAs that have been shown to induce apoptosis in neural crest-derived pheochromocytoma cells and immortalized rat Schwann cells (IFRS1) (Ali et al., 2023). The critical detoxifying role of peroxisomal β-oxidation activity is highlighted by X-linked adrenoleukodystrophy (X-ALD), which is caused by inherited defects in the peroxisomal fatty acid transporter, ABCD1 (Uzor et al., 2020). X-ALD results in the accumulation of VLCFAs and is associated with neurocognitive deficits, vision impairment, white matter injury, seizures and motor deficits, as will be discussed later in this review. The end products of peroxisomal β-oxidation, including propionyl-CoA and acetyl-CoA, are shuttled to mitochondria to complete their oxidation to CO_2_ and H_2_O (Wanders et al., 2023). Peroxisomes also contribute to the synthesis of lipid species, including the anti-inflammatory polyunsaturated fatty acid DHA and essential myelin sheath ether phospholipids (Berger et al., 2016; Uzor et al., 2020).

Peroxisomes also contribute to ROS homeostasis [for a comprehensive list of peroxisome biological functions (see Wanders et al., 2023)]. Generation of ROS, such as superoxide and hydrogen peroxide (H_2_O_2_), occurs as a by-product of aerobic metabolism primarily by mitochondria, the ER and peroxisomes (Schrader and Fahimi, 2006). Peroxisomes produce a significant amount of (H_2_O_2_) as a by-product of their metabolic activity (including, but not limited to, fatty acid oxidation, oxidation of D-amino acids and bile acid synthesis) (Schrader and Fahimi, 2006; Fujiki et al., 2022). In order to detoxify these highly reactive molecules, peroxisomes contain an abundance of ROS-scavenging enzymes, including catalase (which breaks down H_2_O_2_ to H_2_O and O_2_), superoxide dismutase, and peroxiredoxins (Schrader and Fahimi, 2006). During tissue ischemia, infection/inflammation and metabolic disorders (including the peroxisome biogenesis disorder, Zellweger syndrome), ROS generation can exceed the cell’s capacity for detoxification, thereby contributing to disease pathophysiology (Cipolla and Lodhi, 2017; Schrader and Fahimi, 2006). Upregulation of peroxisomal oxidative stress in cells by decreasing expression of catalase or use of peroxisome-targeted KillerRed was associated with perturbance of the redox state of mitochondria and mitochondrial fragmentation (Ivashchenko et al., 2011). ROS also contribute to intracellular signaling cascades under normal physiological conditions. Peroxisomal ROS has been shown to regulate pexophagy through the activation of tuberous sclerosis proteins, specifically TSC2, and inhibition of mTOR complex 1 (Zhang et al., 2013).

## Peroxisomes and immunity

Peroxisomes play a vital role in innate immunity by synthesizing immunomodulatory signaling molecules and by contributing to antiviral and antibacterial signaling cascades. Dixit et al. (2010) were the first to elucidate the antiviral functions of peroxisomes. During host cell invasion, viral products (viral nucleic acids, proteins and lipids) are detected by pattern recognition receptors (PRRs) found on host cell membranes and within the host cell cytosol. When viral nucleic acids are detected by cytosolic PRRs including RIG-I-like receptors (RLRs) a conformational change in these receptors promotes their interaction with mitochondria- and peroxisome-localized MAVS. Expression of interferon types I and III are induced downstream of MAVS activation (Dixit et al., 2010; Odendall et al., 2014). RLR-mediated type III interferon response has been observed in cells infected with Reovirus, Dengue and Sendai viruses (Odendall et al., 2014). The lysosome-, endosome- and peroxisome-localized histidine and di-tripeptide transporter SLC15A3 has also been reported to interact with MAVS and the STING pathway to enhance interferon types I and III responses and thereby to inhibit Chikungunya and HSV-1 virus replication (He et al., 2018).

Especially relevant to neurotropic viral responses, MAVS receptor is essential for full activation of microglial anti-viral responses, such as cytokine release (Gern et al., 2024). Transgenic mice deficient in MAVS infected with the neurotropic virus, vesicular stomatitis virus, showed increased viral neuroinvasion, more non-microglial myeloid cell detection, and increased inflammatory cytokine expression in brain tissue from knockout mice compared to controls, suggesting microglial MAVS is important for viral detection and protective antiviral immunity.

Seminal studies by Di Cara et al. (2017) have demonstrated a critical role for peroxisomes in the host response to bacterial infection. Peroxisomes were observed to be closely associated with phagosomes and were found to be required for phagocytosis by coordinating phagosome formation through their modulatory actions on the assembly of the actin network (Di Cara et al., 2017). Additionally, these authors demonstrated that peroxisomal metabolism of reactive oxygen and nitrogen species directly regulates NF-κB signaling in response to bacterial infection (Di Cara et al., 2017).

Peroxisomes have also been implicated in regulating inflammasomes, which are pro-inflammatory complexes implicated in many neuroinflammatory conditions, although a direct link between peroxisome function and inflammasome activation remains to be elucidated. Inflammasomes are multi-protein complexes that stimulate caspase 1 activation, resulting in cleavage and activation of pro-IL-1β, proIL-18 and gasdermin D (GSDMD) (Zheng et al., 2020). Cleaved GSDMD forms pores in cell membranes, facilitating release of IL-1β and IL-18 and promoting an inflammatory regulated/programmed cell death termed pyroptosis. The peroxisome-localized protein FAMIN has been identified as a regulator of inflammasome activation and antibacterial host response through its ability to complex with fatty acid synthase and control fatty acid oxidation (Cader et al., 2016). It is important to note that subsequent studies have not localized FAMIN to peroxisomes (Vijayan et al., 2017). Pluripotent stem cells derived from patients with X-ALD were found to over produce 25-hydroxycholesterol, and stereotactic injection of 25-hydroxycholesterol into mouse corpus callosum was found to induce NLRP-3-dependent oligodendrocyte cell death and microglial activation (Jang et al., 2016). Additionally, treatment with 4-phenylbutyrate, a peroxisome proliferating agent, in cuprizone-exposed mice prevented induction of inflammasome-associated transcripts *NLRP3* and *IL-1β* and reduced cuprizone-induced demyelination in the corpus collosum (Roczkowsky et al., 2022).

The immunomodulatory properties of peroxisomes are also due in part to the metabolic products of this organelle (Di Cara et al., 2023). Peroxisomes contribute to the synthesis of polyunsaturated fatty acids relevant for immune function, EPA and DHA, that are precursors to various anti-inflammatory and proinflammatory signaling lipids, including resolvins, lipoxins, protectins, prostaglandins, prostacyclins, thromboxanes and leukotriens (Di Cara et al., 2023; Savary et al., 2012). DHA and EPA have been implicated both in immune cell activation through their binding to G-protein coupled receptors on the surface of various innate and adaptive immune cells (including dendritic cells, monocytes, macrophages, neutrophils, lymphocytes and natural killer T-cells) and in the resolution of the inflammatory process by binding to nuclear receptors (including PPARγ, RXR and HNF) (Hidalgo et al., 2021; Alvarez-Curto and Milligan, 2016). Relevant to the antimicrobial actions of peroxisomes, DHA is essential for phagosome formation in murine bone marrow-derived macrophages (Di Cara et al., 2017). In lipopolysaccharide (LPS)-exposed murine macrophages, treatment with 4-phenylbutyrate improved the LPS-induced inflammatory response, which was dependent on peroxisome-derived DHA (Vijayan et al., 2017).

A class of peroxisome-derived glycerophospholipids known as ether lipids, which include plasmalogens, are a major constituent of cellular lipid membranes and contribute to many vital cellular signaling pathways (Wanders et al., 2023). Ether lipids are characterized at the sn-1 position in the glycerol backbone by either an ether linkage (alkyl lipids) or a vinyl ether linkage (plasmalogens), which is believed to impart antioxidant properties to plasmalogens, as this bond is easily oxidized when exposed to oxidizing reagents in the environment, such as peroxyl radicals and UV light, while sparing nearby critical phospholipid components (Braverman and Moser, 2012). With regards to immunity, plasmalogens exert both proinflammatory and anti-inflammatory properties due in part to their fatty acid tails, which are enriched in polyunsaturated fatty acids such as arachidonic acid and DHA (Wanders et al., 2023). Peroxisome-derived ether lipids have also been implicated in immune cell development and survival. Inhibiting ether phospholipid synthesis in mice resulted in the depletion of neutrophils (Lodhi et al., 2015), while treatment of HL-60 cells knocked down for fatty acid synthase with ether lipids improved cell viability *in vitro*. Peroxisome-derived lipids, including ether lipids, are also essential for the development of iNKT cells in the thymus, which involves CD1d receptor on the surface of iNKT cells recognizing lipid self antigens (Facciotti et al., 2012). While immunodeficiency is not a core clinical phenotype of ZSD, there have been case reports of ZSD patients with associated lymphopenia and thymic hypoplasia or atrophy (Gilkrist et al., 1974; Lucaccioni et al., 2020).

## Peroxisomes and the CNS

Peroxisomes have been localized to all cell types within the CNS and contribute to brain health and disease, in part, due to their role in ROS homeostasis, metabolic products and contributions to innate immunity (Berger et al., 2016; Di Cara et al., 2019; Ivashchenko et al., 2011). Peroxisomes generate key myelin lipid components including plasmalogens that are critical for the maintenance of healthy white matter (Berger et al., 2016). Indeed, an oligodendrocyte-specific *Pex5* knockout mouse model exhibited progressive subcortical demyelination and axonal injury without concomitant oligodendrocyte cell death (Kassmann et al., 2007). An 80% reduction in brain plasmalogen levels was detected in *Pex5* knockout mice (Kassmann et al., 2007). As previously discussed, peroxisomes are critical for the synthesis of various polyunsaturated fatty acids such as DHA that have been reported to contribute to neurite outgrowth and synaptogenesis in rat hippocampal neural cells (Calderon and Kim, 2004; Cao et al., 2009; Cao et al., 2005). DHA and EPA have also been found to facilitate the differentiation of neural stem cells into neurons *in vitro* and *in vivo* (Katakura et al., 2013; Katakura et al., 2009; Kawakita et al., 2006). In addition to their synthetic functions, peroxisomes degrade neurotoxic metabolites, such as VLCFAs, that have been found to induce cell death in cultured primary oligodendrocytes and to upregulate markers of oxidative stress in a cultured SKN neuronal cell line (Berger et al., 2016; Wanders et al., 2023; Schönfeld and Reiser, 2016; Zarrouk et al., 2012). The ability of peroxisomes to degrade D-serine and D-aspartate is also considered to modulate synaptic transmission and excitotoxicity (Berger et al., 2016).

Of particular interest is the role of peroxisomes in microglia, the resident brain macrophages in the CNS. Microglial-specific deletion of the gene for peroxisomal β-oxidation enzyme multifunctional protein-2 (*Mfp2*) in a transgenic mouse model resulted in microgliosis in the cortex and brainstem and was associated with a proinflammatory microglial phenotype (Beckers et al., 2019). Similarly in the BV-2 murine microglial cell line, knock out of the gene encoding peroxisomal acyl-CoA oxidase 1 (*Acox1*) enzyme responsible for the first step in peroxisomal β-oxidation resulted in impaired proliferation of microglia, accumulation of VLCFAs, and upregulation of *Il-1b* and *Trem2* mRNA amounts (Raas et al., 2019). More recently, a study examining the effects of *Abcd1*, *Abcd2* and *Acox1* knockouts in BV-2 microglia found that *Abcd1-* and *Abcd2-*deleted cells exhibited lower phagocytic capacity for myelin sheath debris, whereas *Acox1*-deleted cells showed increased phagocytosis of myelin (Tawbeh et al., 2023). Genes encoding for proinflammatory cytokines, T-cell stimulation, antigen presentation and phagocytosis were also altered at baseline and in response to LPS stimulation in knockout cells compared to wild-type cells. Thus, peroxisomes may have important roles in microglial activation, phagocytosis and pro-inflammatory responses.

In addition to their important brain functions, peroxisomes are critical for retinal health and are expressed in cells of the retinal pigmented epithelium, interneurons, ganglion cells and photoreceptor cells (Das et al., 2021; Chen et al., 2022). Their function in retinal homeostasis include their ability to degrade VLCFAs and generate DHA as well as very long chain poly unsaturated fatty acids, although these processes is not yet fully understood in the retina. Regardless, retinopathy is a hallmark of disorders of peroxisome function and abundance, including Zellweger spectrum disorders and x-linked adrenoleukodystrophy (Das et al., 2021; Omri et al., 2025; Yergeau et al., 2023). In peroxisome deficient PEX1-G844D transgenic mice, accumulation of Iba-1 immunopositive cells were detected in the subretinal space (Omri et al., 2025). Additionally, upregulation of inflammatory-related genes and decreased levels of DHA were observed in Mfp2 knockout mouse model (Das et al., 2021).

## Inherited disorders of peroxisome function

Congenital disorders affecting peroxisome function and abundance, as demonstrated by the PBDs, highlight the important functions of peroxisomes in brain health and disease (Fujiki et al., 2022). Zellweger spectrum disorders (ZSDs), including archetypal Zellweger syndrome, neonatal adrenoleukodystrophy (NALD) and infantile Refsum disease (IRD), are PBDs caused by mutations in peroxin genes (*PEX1*, *PEX2*, *PEX3*, *PEX5*, *PEX6*, *PEX10*, *PEX11β*, *PEX12*, *PEX13*, *PEX14*, *PEX16*, *PEX19* or *PEX26*). Zellweger Syndrome is the most clinically disabling of the ZSDs and the most severe phenotypes can be associated with death in infancy. As newborns, these patients present with severe neurological disability, including profound hypotonia, seizures, visual impairment and sensorineural deafness (Braverman et al., 2013). Neuropathological analyses of these patients include abnormalities in both gray and white matter. Cortical pathology is believed to be secondary to impaired neuronal migration, resulting in a decreased number of neurons in the outer cortical layers (layers II and III) (Powers and Moser, 1998). There is also a decrease in the volume of white matter in these patients as observed by both MRI and histology, although it is unclear if this results from impaired myelination, deficiencies in maintaining myelin mass, demyelinating injury, or a combination of all three processes (Berger et al., 2016; Barkovich and Peck, 1997; Baes and Aubourg, 2009; Powers, 1995).

Less severe phenotypes of ZSDs, which include NALD and IRD, are associated with later diagnosis in childhood or even in early adulthood if symptoms and signs are limited (Berger et al., 2016; Fujiki et al., 2022). Childhood and later onset profile of ZSDs includes retinopathy and sensorineural deafness, failure to thrive, developmental delay, cerebral ataxia, and/or peripheral neuropathy. NALD is associated with an early onset and progressive leukodystrophy, which affects both the cerebrum and cerebellum. Patients with late-onset white matter disease display a unique phenotype characterized by minimal distinctive early features and normal development within the first year of life, but subsequently patients develop neurological regression and demyelinating brain injury in childhood or early adulthood (Barth et al., 2001). Interestingly, a study by Barth et al. (2004) investigating the disease phenotype of 25 patients with ZSD was unable to detect a relationship between the severity of leukoencephalopathy and associated *PEX* mutation.

Intriguingly, case studies of PEX16 mutations have also documented atypical Zellweger spectrum disorder phenotypes including dystonia, cerebellar ataxia, corticospinal tract degeneration, peripheral neuropathy and spasticity, with normal levels of VLCFAs (Kumar et al., 2018; Ebberink et al., 2010). Reduced peroxisome density, altered morphology, and impaired catalase activity were observed in cultured neural stem cells derived from olfactory mucosa of affected patients (Kumar et al., 2018).

In addition to PBDs, there are several monogenic disorders that affect single peroxisome enzymes, including X-ALD that is caused by mutation in the gene encoding the peroxisomal VLCFA transporter (*ABCD1*). This disorder most often presents in children of early school age and is typified by declining school performance and behavior disruption. Patients progress to develop deafness, visual impairment, cognitive decline, seizures and spastic limb paresis, with death occurring within a few years if no treatment is initiated (Poll-The and Gärtner, 2012). Initial white matter lesions in this condition are usually localized within the splenium of the corpus callosum, progressing to involve subcortical white matter within the parietal and occipital lobes. Due to the nature of the genetic mutation, increased plasma concentrations of VLCFAs is a sensitive test for detecting this condition in male patients, although up to 15% of females with X-ALD have normal VLCFA amounts in plasma (Moser et al., 2007).

The importance of CNS peroxisomes in neurological health and injury is also confirmed by mouse knockout models. Genetic deletion of *Pex2*, *Pex5,* or *Pex13* in mice causes profound and global peroxisome deficiency, and is associated with growth delay, severe hypotonia, and death shortly after birth, similar to Zellweger syndrome in humans (Baes et al., 1997; Faust and Hatten, 1997; Maxwell et al., 2003). Pathological analysis of these mice revealed reduced neocortical plate thickness and cerebellar malformation, believed to be secondary to impaired neuronal migration. Neural cell-specific deletion of *Pex5* in mice resulted in ablation of functional peroxisomes from oligodendrocytes, astrocytes and neurons, but not from microglia, which are yolk-derived, unlike other neural cell types (Krysko et al., 2007). These knockout mice exhibited progressive motor impairments and lethargy with marked global cerebral hypomyelination, neuronal cell loss and microgliosis detected 2–3 weeks postnatally (Bottelbergs et al., 2012). Similarly, decreased amounts of plasmalogens, microgliosis, and impaired cortical layer formation were observed in *Pex13* neural cell knockout mice (Muller et al., 2011). These studies highlight the severe consequences of peroxisomal dysfunction on neural cell functions.

## Peroxisomes in neuroinflammation-associated disorders

The term “neuroinflammation” describes the unique immune response within CNS tissues due to inflammation-provoking injuries (e.g., trauma, ischemia, hypoxia, pathogenic protein deposition), endogenous cellular stressors (e.g., mitochondrial dysfunction and oxidative stress) or invasion by foreign species (e.g., viruses, bacteria, fungi, toxins). The role of peroxisomes in neuroinflammation is once again demonstrated by genetic conditions resulting in peroxisome ablation. Studies utilizing oligodendrocyte-specific *Pex5* knockout mice found progressive demyelinating injury associated with upregulation of inflammatory cytokines, reactive gliosis, and infiltration of CD8 + T-cells into the brains of knockout mice (Kassmann et al., 2007). The progressive demyelination observed in brains of patients with X-ALD is associated with increased VLCFAs, gliosis and increased proinflammatory cytokines (IL-15, IL-12p40, CXCL8, CCL11, CCL22, IL-4) detected in the blood, in demyelinating plaques (including increased IL-1β, TNF-α, IFN-*γ*), and in cerebrospinal fluid (CSF) (IL-8, IL-1ra, MCP-1, MIP-1b) of affected patients relative to controls (Lund et al., 2012; Weinhofer et al., 2023; Schlüter et al., 2012). Additionally, blood–brain barrier disruption and infiltration of circulating macrophages have been observed in patients with childhood adrenoleukodystrophy (a severe form of X-ALD) (Berger et al., 2014). Within CNS tissues from mice knocked out for the *Mfp2* gene, extensive microgliosis and increased mRNA levels of *Tnf-α, Il-1b, Il-6, C1q*, and *Tlr2* were detected (Verheijden et al., 2013). Unsurprisingly then, peroxisome dysfunction has been implicated in the pathophysiology of numerous neuroinflammatory brain conditions, including multiple sclerosis (the prototypic neuroinflammatory disorder), neurotropic viral infections (i.e., encephalitis), and neurodegenerative conditions with an inflammatory component, such as Alzheimer’s disease (Table 1).

**Table 1:** Peroxisome impairments in neurological disorders.

**Disease**	**Observed Peroxisome Impairment and Relevant References**
Multiple sclerosis	Decreased plasmalogen levels and increased VLCFA levels in MS patients and animal models of MS (Yanagihara and Cumings, 1969; Singh et al., 2004; Senanayake et al., 2015). Decreased PMP70 protein levels (Gray et al., 2014) and suppressed *PEX3* and *PEX5L* transcripts in brain tissue from MS patients (Roczkowsky et al., 2022). Suppressed *Abcd1*, *Cat* and *Pex5L* mRNA transcript levels and decreased catalase protein levels in brain tissue of cuprizone-exposed mice (Roczkowsky et al., 2022); decreased catalase and DHAP-AT levels in spinal cord of EAE mice (Singh et al., 2004).
Alzheimer’s disease	Suppressed plasmalogens, decreased DHA and elevated VLCFA levels in brain tissue from AD patients (Söderberg et al., 1991; Ginsberg et al., 1995; Lukiw et al., 2005; Astarita et al., 2010; Kou et al., 2011; Lizard et al., 2012). Increased PMP70 immunostaining in neuronal cell bodies and decreased PMP70 immunostaining in neuronal processes in brain tissue from AD patients (Kou et al., 2011). Increased PMP70 and PEX5 immunostaining and decreased catalase and GPX-1 immunostaining in neocortex and hippocampus of transgenic (Tg2576) mice (Cimini et al., 2009). Decreased catalase activity and increased cell death of primary rat hippocampal neurons exposed to β-amyloid (Santos et al., 2005).
Parkinson’s disease	Suppressed DHA and arachidonic acid levels in brain tissue of PD patients (Fabelo et al., 2011); decreased plasmalogen levels in brain and blood of PD patients (Dragonas et al., 2009; Miville-Godbout et al., 2016). In mouse models of PD: plasmalogen-dependent decrease in striatal dopamine markers (Miville-Godbout et al., 2016); decreased catalase activity and suppressed *Cat*, *Pex14* and *Abcd3* mRNA levels in mice overexpressing-synuclein (Yakunin et al., 2014).
Amyotrophic lateral sclerosis	Mutations in the gene for peroxisomal D-amino acid oxidase (R199W D-AA) detected in familial form of ALS (Mitchell et al., 2010). Pathway enrichment analysis identified peroxisome pathways as significantly associated with ALS (Du et al., 2018). Impaired cholesterol metabolism detected in ALS patients (Abdel-Khalik et al., 2017).

### Multiple sclerosis

Multiple sclerosis (MS) is a progressive neurological disease characterized by inflammatory demyelination within the CNS (Filippi et al., 2018). Due to the integral roles of peroxisomes in maintaining myelin and modulating immune responses, peroxisome dysfunction has been implicated in the pathophysiology of MS. Over half a century ago, a study by Yanagihara and Cumings (1969) established a link between perturbed peroxisome function and MS, reporting that levels of plasmalogens were decreased in white matter from CNS samples of patients who died from MS. More recently, the link between MS and peroxisomes has been better elucidated. Decreased peroxisome abundance (as measured by PMP70 immunostaining) and increased VLCFA levels were observed in the gray matter of brains from patients with MS versus patients without MS (Gray et al., 2014). Elevated VLCFA levels were detected in the serum of patients with various forms of MS (relapsing remitting, primary progressive, secondary progressive), and VLCFA levels correlated with duration of illness in patients with relapsing remitting MS (Senanayake et al., 2015). Similarly, elevated levels of VLCFAs and decreased levels of plasmalogens were observed in brains from experimental autoimmune encephalomyelitis (EAE) mice, a model for MS (Singh et al., 2004). Peroxisomal catalase and DHAP-AT levels were also significantly reduced in the spinal cords of mice with EAE, which was prevented by treatment with lovastatin. Recent studies by Roczkowsky et al. (2022) have identified reduced peroxisomal transcripts and protein levels, including PMP70, in white matter from MS patients compared to non-MS controls. In a cuprizone-exposure model of MS, cuprizone induced severe demyelinating injury as detected by reduced myelin immunolabeling and suppressed critical peroxisome transcripts, including *Abcd1*, *Cat* (catalase), and *Pex5L*, which was prevented by treating cuprizone-exposed mice with the peroxisome proliferating agent, 4-PBA. Importantly, 4-PBA was also protective against cuprizone-induced neurobehavioral deficits. Additionally, many studies have demonstrated a protective effect of peroxisome proliferator-activated PPAR agonists in animal models of MS due, in part, to their roles in modulating both innate and adaptive immune cells (Racke et al., 2006; Ferret-Sena et al., 2018).

### Alzheimer’s disease

Alzheimer’s disease (AD) is an irreversible neurodegenerative disorder characterized by progressive deterioration in memory and executive functioning (DeTure and Dickson, 2019). It is the most prevalent form of dementia world-wide, affecting over 55 million people globally according to the World Health Organization. Although AD is not traditionally considered a neuroinflammatory condition, neuroinflammation appears to play a key role in the pathogenesis of AD beyond a passive response to the accumulation of β-amyloid plaques and neurofibrillary tangles (Heneka et al., 2015; Heneka et al., 2024). Changes in the inflammatory profiles of microglia have been correlated with AD disease progression (Heneka et al., 2024; Deczkowska et al., 2018). Moreover, increased TPSO ligand binding in brain during positron emission tomography (PET) studies, which correlates with glial cell activation, is associated with greater neurocognitive decline and increased brain atrophy among patients with AD (Kreisl et al., 2013; Kreisl, 2017). Additionally, GWAS studies have identified genetic risk factors, which are highly expressed in microglia and important for innate immunity, such as TREM2 and HLA-DQA1 (Heneka et al., 2025). Perturbations in lipids have also been detected in brains of patients with AD, including depletion of plasmalogens in post-mortem brain samples of patients with AD (Kou et al., 2011; Ginsberg et al., 1995; Lizard et al., 2012) and accumulation of peroxisome-specific VLCFAs in autopsy cortical brain samples of patients with AD (Kou et al., 2011). Decreased levels of plasmalogens and increased levels of VLCFAs were associated with the presence of neurofibrillary tangles in brain samples (Kou et al., 2011). Concentrations of the peroxisomal metabolite DHA are reduced in the hippocampus, frontal cortex and temporal cortex in post-mortem samples from patients with AD versus controls (Söderberg et al., 1991; Lukiw et al., 2005). Interestingly, the ratio of DHA to α-linolenic acid in temporal cortex and mid-frontal cortex of patients with AD correlated with results from the Mini Mental State Examination (Astarita et al., 2010), and treatment with DHA improves the burden of amyloid plaques, neurofibrillary tangles and neuroinflammation in the brains of animal models of AD (Pan et al., 2015).

Studies directly measuring peroxisome abundance are few in AD patients or in preclinical studies of AD. Kou et al. (2011) demonstrated increased peroxisomal volume in neuronal soma within the gyrus frontalis in post-mortem brain samples of patients with advanced AD by immunohistochemical staining for the peroxisome marker, PMP70. Additionally, in the same cohort of AD patients, neuronal processes containing abnormally phosphorylated tau protein demonstrated reduced PMP70 immunostaining, suggesting impaired peroxisome protein trafficking in patients with AD (Kou et al., 2011). In an animal model of AD using Tg2576 transgenic mice, PMP70 and PEX5 immunohistochemical and immunofluorescence labeling were increased in the hippocampus and neocortex of transgenic mice versus wild-type mice, whereas catalase and glutathione peroxidase (GPX1) immunostaining were decreased in the hippocampus and increased in the neocortex of transgenic versus wild-type mice (Cimini et al., 2009). In a study by Santos et al. (2005) treatment of cultured rat primary hippocampal neurons with the peroxisome proliferator Wy-14,643 prevented a decrease in peroxisome numbers due to β-amyloid exposure, preserved catalase activity, and protected cells from β-amyloid-induced cell death.

### Parkinson’s disease

Parkinson’s disease (PD) is an α-synucleinopathy characterized by death of dopaminergic nigrostriatal neurons in the substantia nigra leading to progressive motor impairment and eventual neurocognitive impairment and autonomic dysfunction (Morris et al., 2024). Although the role of neuroinflammation in PD is not yet well understood, studies have observed microgliosis in autopsy samples of patients with PD and in patients with rapid eye movement (REM)-sleep behavior disorders, suggesting microglial activation may contribute to disease pathology (Harms et al., 2021; Isik et al., 2023). Analysis of lipid rafts from autopsy brain samples from PD patients showed reductions in the peroxisomal lipid products DHA and arachidonic acid compared to control patients (Fabelo et al., 2011). Studies have found decreased levels of plasmalogens in the brain and blood of patients with PD, and treatment with the DHA-plasmalogen precursor PPI-1011 improved plasmalogen levels and reversed loss of striatal dopamine markers (dopamine, dopamine transporter, vesicular monoamine transporter-2) in a MPTP-treated mouse model of PD (Miville-Godbout et al., 2016; Dragonas et al., 2009). Transgenic mice overexpressing α-synuclein (A53T α-syn mice) exhibited decreased catalase activity and lower levels of *Cat, Pex14,* and *Abcd3* mRNAs in whole brain tissue compared to wild-type controls (Yakunin et al., 2014). Much like the neurological illnesses discussed previously, PPAR agonists such as rosiglitazone, pioglitazone and fenofibrate have demonstrated neuroprotective effects in animal models of PD (Breidert et al., 2002; Barbiero et al., 2014; Lee et al., 2019), but these studies did not measure peroxisome abundance or function and will not be elaborated on further.

### Amyotrophic lateral sclerosis

Neuroinflammation has been proposed to contribute to the pathogenesis of amyotrophic lateral sclerosis (ALS) (Arnoux and Dupuis, 2021). ALS is characterized by degeneration of spinobulbar motor neurons and corticospinal neurons. A large-scale genome-wide association study in patients with ALS identified several peroxisomal genes and pathways associated with this uniformly lethal disorder (Du et al., 2018). Additionally, altered levels of cholesterol precursors and products were observed in the CSF of patients with ALS, suggesting potential peroxisome dysfunction (Abdel-Khalik et al., 2017). Mutations in the peroxisomal D-amino acid oxidase (DAO) gene (R199W D-AA) has been detected in patients with familial amyotrophic lateral sclerosis, and overexpression of R199W D-AA in motor neurons led to cell death (Mitchell et al., 2010).

### Neurotropic viruses

As discussed earlier, peroxisomes have been implicated in mediating host antiviral responses to a range of viruses (Table 2). Relevant to the discussion of neuroinflammation, peroxisome dysfunction has been implicated in the pathophysiology of several neurotropic viruses causing neuroinflammation, such as SARS-CoV-2, the virus responsible for the COVID-19 pandemic. Neurological disorders are associated with COVID-19, such as anosmia, ageusia, seizures and encephalitis, and although the mechanisms underlying COVID-19-associated neurological symptoms have not been fully elucidated, neuroinflammation appears to play a pivotal role (Balcom et al., 2021; Lee et al., 2021; Colombo et al., 2021). The host cell receptors for SARS-CoV-2, including ACE-2 and the co-receptor neuropilin-1, are expressed throughout the CNS, including on astrocytes and neurons (Chen et al., 2020; Davies et al., 2020), and SARS-CoV-2 genomes have been detected in brain tissues from humans and animal models (Matschke et al., 2020; Stein et al., 2022). Peroxisome dysfunction has been observed in both *in vivo* and *in vitro* models of SARS-CoV-2 infection. Studies using affinity-purification mass spectrometry identified several peroxins, including PEX3 and PEX11β, that bind *in vitro* to the SARS-CoV-2 protein, ORF14 (Gordon et al., 2020). *In vitro* infection of a neuroblastoma cell line (SK-N-SH) resulted in reduced peroxisomal abundance in infected cells, which was hypothesized to be due in part to physical interaction between ORF14 and PEX14 (Knoblach et al., 2021). A recent study by Roczkowsky et al. (2023) found reduced peroxisomal transcripts and proteins (including PEX3, PMP70 and PEX14) and robust induction of cytokine transcripts (including *Il-8* and *Cxcl10*) and protein levels (IL-18 and GM-CSF) in brain samples from COVID-19 patients compared to other disease controls. Furthermore, hamsters infected intranasally with SARS-CoV-2 showed detectable virus in the olfactory bulb at 7 days post infection, and a sustained neuroinflammatory response in the cortex that was accompanied by decreased catalase protein levels relative to uninfected hamsters (Roczkowsky et al., 2023). In an animal model of post-acute sequelae of COVD-19, treatment of mice with 4-PBA significantly reduced mortality, further supporting the role of peroxisome dysfunction in COVID-19 (Wei et al., 2025). As peroxisomes are known to mediate interferon responses, peroxisome suppression may be a virus-induced mechanism for host immune evasion.

**Table 2:** Peroxisome impairments in neurotropic viral infection.

**Virus**	**Observed Peroxisome Impairment**	**Proposed Mechanisms**
SARS-CoV-2	Suppressed PEX3, PMP70 and PEX14 immunolabeling *in vitro* (Knoblach et al., 2021). Suppressed *PEX3*, *PEX11β*, *PEX5L* and *PEX14* mRNA levels and decreased PMP70 and PEX14 protein levels in brain tissue from COVID-19 patients versus controls (Roczkowsky et al., 2023). Suppressed *Pex3* mRNA and catalase protein in brains from infected hamsters (Roczkowsky et al., 2023).	Binding of viral protein ORF14 to peroxins PEX3, PEX11β or PEX14 (Gordon et al., 2020; Knoblach et al., 2021). Upregulation of Wnt/β-catenin signaling (Xu, Elaish et al. 2024). Cytokine-induced suppression of peroxin genes (Roczkowsky et al., 2023).
Human Pegivirus	Suppressed *ABCD3*, *PEX11β*, *IRF1* and *IRF3* mRNA levels in brain tissues of Pegivirus-positive patients (Doan et al., 2021). Decreased *ABCD1*, *ABCD3*, *PEX7*, *PEX11β*, and *MAVS* transcripts in infected human astrocytes and microglia (Doan et al., 2021).	Not known.
West Nile virus	Suppressed peroxisomal numbers and reduced PEX19 immunostaining *in vitro* (You et al., 2015).	Binding of viral capsid proteins to PEX19 (You et al., 2015).
Dengue virus	Suppressed peroxisomal numbers and reduced PEX19 immunostaining *in vitro* (You et al., 2015).	Binding of viral capsid protein to PEX19 (You et al., 2015).
Zika virus	Suppressed peroxisome abundance and decreased PEX11β and PEX19 protein levels in infected human astrocytes and U251 cells (Wong et al., 2019).	Binding of viral capsid protein to PEX19 (Wong et al., 2019).
CMV	Induced fragmentation of peroxisomes and suppressed peroxisome-dependent antiviral signaling (Magalhães et al., 2016).	Interaction between viral protein vMIA and Pex19 (Magalhães et al., 2016).
HSV-1	Suppressed peroxisomal MAVS signaling (Zheng and Su 2017).	Unknown mechanism, mediated by virally encoded protein VP16 (Zheng and Su 2017).
EBV	Suppressed ABCD1 and ABCD2, resulting in increased VLCFA levels in infected human B-cells (Weinhofer et al., 2022). Upregulation of PEX19 and altered lipid metabolism in infected PBMCs (Indari et al., 2023).	Induction of miRNAs (miR-9-5p and miR-155) (Weinhofer et al., 2022).
HIV-1	Reduced peroxisome abundance and decreased PEX13, PEX7, PEX2 and PEX11β protein levels (Xu et al., 2020).	HIV-associated protein, Vpu, induced upregulation of peroxin-targeting miRNAs (Xu et al., 2020).

Several members in the flavivirus family of positive-RNA strand viruses, specifically Pegivirus, Dengue, West Nile, and Zika viruses, have been implicated in both neurotropism and peroxisome suppression. Pegivirus infects human astrocytes and microglia *in vitro* (Doan et al., 2021) and is associated with fatal leukoencephalitis in immunocompromised patients (Balcom et al., 2018; Doan et al., 2020; Scheibe et al., 2025). Several peroxisomal and type I interferon-related transcripts (including *ABCD3*, *PEX11β*, *IRF1* and *IRF3*) were suppressed in brain samples from patients with Pegivirus brain infection (Doan et al., 2021). These results were replicated *in vitro* with Pegivirus infection of human glial cells, demonstrating suppression of peroxin and interferon gene transcript levels (including transcripts for MAVS) compared to uninfected cells (Doan et al., 2021). Investigation of host cell protein interaction with viral proteins produced by West Nile and Dengue viruses revealed an interaction between the capsid proteins of these viruses and PEX19 (You et al., 2015). Infection of A549 cells with West Nile or Dengue viruses resulted in reduced PEX19 immunostaining and reduced peroxisome abundance (You et al., 2015). Studies by the Hobman group have demonstrated that Zika virus suppresses peroxisomes in infected human astrocytes and U251 cells, and overexpression of *PEX11β* in U251 cells significantly reduced Zika viral titers by over 80%, demonstrating the importance of peroxisomes in the antiviral response to this pathogen (Wong et al., 2019).

Several members of the Herpesviridae family have also been found to avoid peroxisome-mediated host immune responses through unique mechanisms (Ferreira et al., 2019). By encoding the protein vMIA, cytomegalovirus (CMV) is capable of evading the cellular antiviral response in infected mouse embryonic fibroblasts (Magalhães et al., 2016). vMIA was shown to bind directly to PEX19 and interact with viral MAVS, thereby inhibiting downstream signaling (Magalhães et al., 2016). Herpes simplex virus 1 (HSV-1) has also been found to suppress the peroxisomal MAVS response in infected cells through the actions of viral Magalhães, Ferreira et al. 2016protein VP16 (Zheng and Su, 2017). In contrast, Epstein–Barr virus (EBV), a gammaherpes virus associated with MS (Bjornevik et al., 2022), has been shown to influence peroxisome functions (Weinhofer et al., 2022; Indari et al., 2023).

The neurotropism of human immunodeficiency virus type 1 (HIV-1) is well known and associated with neuroinflammation and neurocognitive dysfunctions in a subset of HIV-infected patients (Gelman, 2015). Peroxisomes play an important role in detecting HIV-1 genomic RNA though the RIG-I and MAVS pathway (Berg et al., 2012). Reduced peroxisome abundance and suppressed levels of multiple peroxins were observed in brain tissues from patients with HIV-associated neurocognitive disorders (HAND) and in HIV-1-infected HeLa cells (Xu et al., 2017). Intriguingly, the mechanism of peroxisome suppression by HIV-1 appears to be related to HIV-induced miRNAs, as several miRNAs relevant to peroxisome biogenesis were identified in brains from patients with HAND, and transfection of HEK293T cells with these miRNAs negatively regulated expression of *PEX* mRNAs (Xu et al., 2017). Subsequent studies have demonstrated that the HIV-associated protein, Vpu, induced expression of peroxisome-associated miRNAs, resulting in depletion of cellular peroxisomes in infected HeLa CD4/CXCR4/CCR5 cells (Xu et al., 2020).

## Conclusion and future directions

The role of peroxisomes in neuroinflammatory disorders is an emerging field, built on years of research focused on the critical roles of peroxisomes in modulating inflammatory responses and contributing to CNS homeostasis. Given the robust neuroinflammatory responses and white matter changes evident in the PBDs, it is not unpredicted that peroxisome dysfunction has been observed in neuroinflammatory conditions such as MS and infections by neurotropic viruses. Many viruses have developed mechanisms for evading peroxisome-mediated immune responses, likely contributing to the inflammatory sequelae caused by these viruses. Nonetheless, the relative contributions of peroxisome dysfunctions and vulnerabilities to neuroinflammation remain unknown for different neural cell types (e.g., microglia, neurons, astrocytes, oligodendrocytes). The utility of analyzing peroxisome constituents and products in CSF as diagnostic biomarkers of neuroinflammation is unclear, although this may be a promising approach to refining diagnostic accuracy and responses to therapies. With the promising results of peroxisome proliferating drugs in preclinical studies of neuroinflammatory conditions, peroxisomes represent novel targets for mitigating the negative aspects of neuroinflammation in common and debilitating neurological disorders, including MS, Parkinson’s and Alzheimer’s diseases.
